# Ground Reaction Force and Valgus Knee Loading during Landing after a Block in Female Volleyball Players

**DOI:** 10.2478/hukin-2014-0008

**Published:** 2014-04-09

**Authors:** David Zahradnik, Jaroslav Uchytil, Roman Farana, Daniel Jandacka

**Affiliations:** 1 Human Motion Diagnostic Center, University of Ostrava, Ostrava, Czech Republic,.

**Keywords:** anterior cruciate ligament, injury prevention, kinetics

## Abstract

A non-contact anterior cruciate ligament (ACL) injury is both a serious and very common problem in volleyball. The aim of the study was to determine the association between stick, step-back, and run-back landings after a block and select risk factors of ACL injuries for female professional volleyball players. The research sample involved fourteen female professional volleyball players. Two force plates were used to determine ground reaction forces. Eight infrared cameras were employed to collect the kinematic data. The one-factor repeated-measures analysis of variance, where the landing type was the factor, was used for comparing the valgus moment and ground reaction force on the right lower limb. ANOVA showed that the type of landing has a main effect on the valgus moment on the right lower limb (F) = 5.96, p = 0.019df = 1.18, partial η^2^ = 0.239 and SP = 0.693). Furthermore, it did not show a main effect on the vertical reaction force on the right lower limb ((F)=2.77, p=0.090, df=1.55, partial η^2^= 0.128 and SP=0.448). The highest valgus moment occurred during the run-back landing. This moment, however, did not have any effect within the first 100 ms after initial contact with the ground, but rather upon the subsequent motion carried out when stepping back off the net. A comparison between a run-back landing and a step-back landing showed relevant higher values of vertical ground reaction forces during the run-back landing.

## Introduction

Non-contact anterior cruciate ligament (ACL) injuries occur primarily in youth, healthy individuals as a result of sudden changes in direction or speed during physical activities (Hewett et al., 2007). This type of knee joint injuries are the most common injuries in volleyball (Decker et al., 2003), and requires medical intervention (Ferretti et al., 1992). A much greater incidence of ACL injuries in volleyball occurs in women (Ferretti et al., 1990). The largest number of ACL injuries occurs during the second decade of life (Griffin, 2007).

A take-off followed by a landing maneuver are the essential fundamentals of techniques regarding attacks, blocks and jump serves in volleyball. During the landing phase it is the ankle, knee and hip joints which are exposed to stress resulting from the action of ground reaction forces. The impact that the reaction forces have on the joints of the lower limbs has already been dealt with by a number of authors (Decker et al., 2003; Ortega et al., 2010; Podraza and White, 2010; Salci et al., 2004; Seegmiller and McCaw, 2003). Hewett et al. (2005) defined risk factors (the abduction angle in the knee joint, dynamic valgus moment and high ground reaction forces) predicting the incidence of ACL injuries in women’s volleyball, basketball and soccer through a prospective study of 205 women.

In order to fulfill the objective of the game, players are forced to reduce the period of landing to a minimum. There are two basic landing situations that occur when blocking a volleyball spike: 1) a successful block; and 2) an unsuccessful block. A successful block is characterized by the completion of a particular play (the ball lands on the opponent’s side of the net after a block) and the player is not subject to time pressure upon landing. Because there is no time pressure on the player during a successful block landing, they have the opportunity to possibly alter their mechanics during landing. An unsuccessful block is characterized by the continuation of the game (after contact with an attempted block the ball continues onto the blocker’s side of the net, where it is continuously played for a following attacking action) and the player is forced, upon landing, to step back away from the net prior to a subsequent attacking action. In this case, the player must react to the game situation and may not have sufficient time to land safely.

There are several landing techniques used by volleyball players in the situations of successful and unsuccessful blocks. Three of these are the stick, the step-back and the run-back landing. Players usually use a stick and a step-back landing in case of a successful block and a run-back landing in case of an unsuccessful block. A stick landing is one in which the landing does not contain a subsequent move. The feet are relatively parallel at the instant of ground contact and the player is able to stand upright without over-balancing. A step-back landing is a modification of the stick landing. The feet are relatively parallel at the instant of ground contact and the player performs a free step away from the net with the right foot immediately upon landing. A run-back landing is a part of the game strategy and results in a run back as quickly as possible away from the net to a distance of approximately 3 m immediately upon landing. The feet are relatively parallel at the instant of ground contact and the player carries out a first step backwards with right lower extremity immediately upon landing. These movements can have an impact on biomechanical factors that present a risk for the occurrence of ACL injuries. The aim of this study was to determine the association between stick, step-back, and run-back landings after block maneuvers and selected risk factors of ACL injuries in female professional volleyball players. It was hypothesized that the type of landing after the block maneuvers in volleyball would affect the valgus moment and the vertical ground reaction force. We anticipated that the run-back landing would increase the values of these two variables.

## Material and Methods

### Participants

Fourteen elite female volleyball players (age 22.5 ± 4.6 years; body height 180.9 ± 0.1 cm; body mass 72.3 ± 8.3 kg) participated in this study. All of them were centre blockers, receiver-hitters and universal players with 2 to 14 years of experience playing in the top league. None of them had any previous history of hip, knee or ankle injuries. At the time of testing, they had no such injuries that would prevent them from performing any physical activities for more than two weeks over the previous six months (Dai et al., 2010). The participants were provided with an oral explanation of the objectives and procedures of the research before being tested. All of the procedures used in this study were approved by the Research Ethics Committee of the Diagnostic Center. The data presented in this paper are from the same participant groups as that reported in Zahradnik et al. (2013).

### Protocol

**Figure 1.**
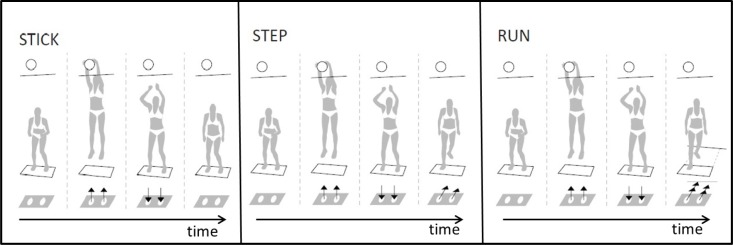
Types of landing

The subjects visited the laboratory on two different days, with an interval of 24 hours, and performed an identical protocol on each day. The experimental setting was based on a real situation of a block maneuver in a volleyball match. The upper edge of the net was at a height of 224 cm above the ground. To normalize the height of the jump, a static volleyball was suspended in the space above the net. The centre of the ball was located 15 cm above the edge of the net and 10 cm behind the edge of the net on the opponent’s side of the court. The data for both dynamic and kinematic analyses were collected from the following three types of landing: stick landing, step-back landing and run-back landing (Figure 1). A stick landing that is not followed by a subsequent movement is defined as a landing maneuver ending with both lower limbs on the ground with an intentional extension of the landing period achieved by a sufficient flexion in the knee joint. A step-back landing is characterized by a free step away from the net performed with the right foot immediately upon landing. A run-back landing is defined as an immediate stepping away from the net to a distance of 3 m that is initiated immediately upon landing by stepping back with the right foot. The subjects were informed of the game situation at the instant of contact (block) with the ball above the net by an acoustic signal. In three situations, the subjects were motivated to execute the landing so that it reflected the situation in a match as close as possible. A warm-up was followed by five practice attempts. Then the subjects had to perform four successful attempts at a stick landing, step-back landing and run-back landing. The randomness of the selection of the individual experimental situations was controlled by the examiner. The subjects were asked after each attempt whether the executed block corresponded with a real situation in a match. If the player’s feedback was negative, the attempt was repeated. The attempt was also repeated when the subjects failed to land on the force plate with the correct foot. A successful attempt was the one in which each foot of the player landed on a separate force platform.

### Experimental set-up

Two force plates (Kistler, 9286 AA, Switzerland) embedded into the floor were used to determine the ground reaction force data at a sampling rate of 1235 Hz. Simultaneously, a motion-capture system (Qualisys Oqus, Sweden) consisting of eight infrared cameras was employed to collect the kinematic data at a sampling rate of 247 Hz. The calibration markers were placed bilaterally on the lateral and medial malleoli, medial and lateral femoral condyles, greater trochanter of the femur and on the shoe over the first and fifth metatarsal heads. The tracking markers were securely positioned to define the trunk (acromion), pelvis (iliac crests, posterior superior iliac spines), thighs and shanks (four light-weight rigid plates holding a quaternion of markers) and shoe (triad of markers on the heel over the calcaneus).

### Data analysis

Both kinetic and kinematic data were processed using Visual3D software (C-motion, Rockville, MD, USA). The scope of movement being monitored started at the moment when the ground reaction force on the force plate (connected with the lower limb) exceeded 20 N and finished either when a step-back landing had a final positive value from the ground reaction force or when a stick landing finished with the return of the subject to the default upright position, i.e. standing up. All lower limb segments were modelled as frusta of right circular cones, while the pelvis and trunk were modelled as cylinders. The local coordinate systems were defined using a standing trial operation. The coordinate data were low-pass filtered using a 4th order Butterworth filter with a 12 Hz cutoff frequency. All data collected from the force plates were low-pass filtered using a 4th order Butterworth filter with a cutoff frequency of 50 Hz. Six degrees of freedom were collected for each segment from motion reflexive markers for the corresponding segment. This was followed by calculating the three dimensional angles in the ankle, knee and hip joints using the Cardan sequence Xyz (Hamill and Selbie, 2004a).

The analysis in this article includes data related to the right lower limb only. Net moments of force in the joints were calculated using the inverse dynamics technique (Hamill and Selbie, 2004b). The ground reaction force was normalized to body weight (in Newtons) and valgus moment was normalized to body weight (in Newtons) and height (in metres) (Hughes et al., 2010). The valgus moment of force on the right lower limb was calculated using the technique by Hamill and Selbie (2004b). The proximal local coordinate system of the knee was oriented so that the valgus moment in the frontal plane of the thigh provided positive numbers and initiated a tendency towards adduction (the movement of the calf toward the middle plane).

### Statistical analysis

All of the analyzed variables were normally distributed (Shapiro-Wilk test). The intraclass correlation coefficient (ICC) was applied for the assessment of the measurements’ reliability (Hopkins, 2000). An ICC value higher than 0.7 was considered to be adequately reliable (Nunnally and Bernstein, 1994). The one-factor repeated-measures ANOVA (factor: a type of landing) was used for comparing the vertical ground reaction force and the valgus moment on the right lower limb. If the Mauchly’s test result was significant, Greenhouse-Geisser corrections were used. This was followed by carrying out Bonferroni pairwise comparisons. The significance of the impact that the types of landing had on the dependent variables was evaluated with the use of the Eta squared index (Cohen, 1973). Since the one-factor analysis of variance was used, we considered that the partial value η^2^ <0.009 represented a trivial effect, partial η^2^ = 0.009–0.0588 a small effect, partial η^2^ = 0.0588–0.1379 a medium effect and partial η^2^ > 0.1379 a large effect (Cohen, 1988). The statistical power of the test (SP) was formulated in accordance with the Cohen’s study (1962). Statistical significance was set for all of the tests at the level of *p* <0.05. All statistical procedures were performed using an IBM SPSS 20.

## Results

The intraclass correlation coefficient (ICC) was determined based on eight repeated measurements of vertical reaction forces and the valgus moment on the right lower limb for all three types of landing. The average value of the ICC in case of the stick landing was 0.799 (ranging from 0.511 to 0.947), in case of the step-back landing it equaled 0.928 (ranging from 0.826 to 0.981) and in case of the run-back landing it was 0.891 (0.736 to 0.971).

The one-factor analysis of variance showed a main effect of a type of landing on the valgus moment on the right lower limb (*F* =5.96, *p* =0.019, *df* =1.18, partial η^2^ =0.239 and *SP* =0.693). The subsequent pairwise comparison using Bonferroni corrections showed that there was a significantly higher valgus moment on the right lower limb during a run-back landing than during a step-back landing (p < 0.042). In addition, no major correlation was verified between a specific landing type and the vertical reaction force on the right lower limb (*F* = 2.77, *p* = 0.090, *df* = 1.55, partial η^2^ = 0.128 and *SP* = 0.448).

## Discussion

The aim of this study was to determine the influence of stick, step-back and run-back types of landing after a block maneuver on the selected risk factors of ACL injuries in professional female volleyball players.

The incidence of anterior cruciate ligament injuries is many times more common among females than males (Shea et al., 2004). Marshall et al. (2007) claimed that there have been only two studies dealing with ACL injuries in volleyball within the last decade. Landing following a take-off is a primary non-contact mechanism causing ACL injuries in women’s volleyball and basketball (Ferretti et al., 1992; Kirkendall and Garrett, 2000). Women, in general, keep a much greater valgus angle during the landing maneuver (Ford et al., 2003; Padua et al., 2004). It has been proven that knee valgus loading increases stress on the anterior cruciate ligament (Lloyd and Buchanan, 2001). According to Hewett et al. (2005), landing in a dynamic valgus position is seen as potentially harmful for the knee joint. Furthermore, the valgus moment in the knee joint is perceived as a predictive factor of ACL injuries (73%). In their study, Hewett et al. (2005) compared nine female athletes who suffered from ACL rupture during sport activities over the course of six months (on average) with female athletes who did not suffer such an injury.

**Figure 2.**
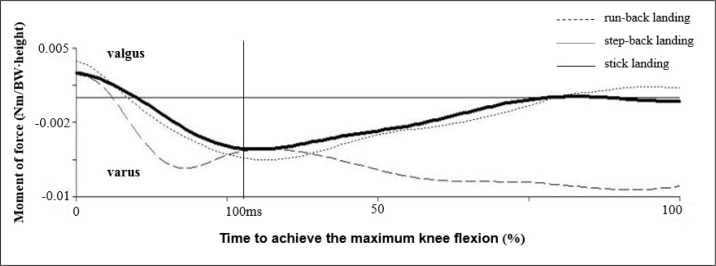
Internal valgus-varus moment in the right knee joint (n=14)

The main conclusion of the study is that when a run-back landing maneuver is followed by immediately stepping away from the net, as opposed to a step-back landing, there is a significantly higher valgus moment in the knee of the right lower limb (Table 1). Our findings are in accordance with the stated hypotheses. During the stance phase (starting from the initial contact with the ground and ending with maximum flexion in the knee joint) it is common for the valgus moment to occur first in all types of landing. This is then followed by the varus moment, which smoothly transforms into the valgus moment in case of the stick landing and step-back landing (Figure 2). It is surprising that the valgus moment (Table 1), in case of the run-back landing, is at its maximum when actively moving away from the net. This phase, however, is not shown in the graphs.

**Figure 3.**
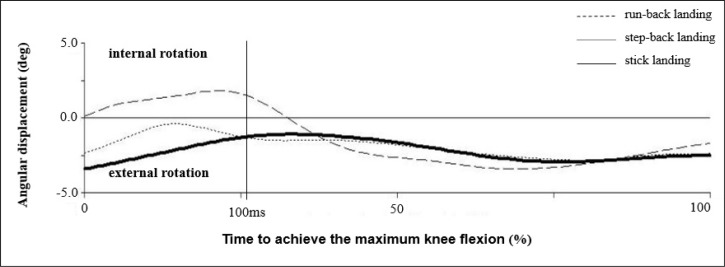
Internal-external rotation in the right knee joint (n=14)

**Figure 4.**
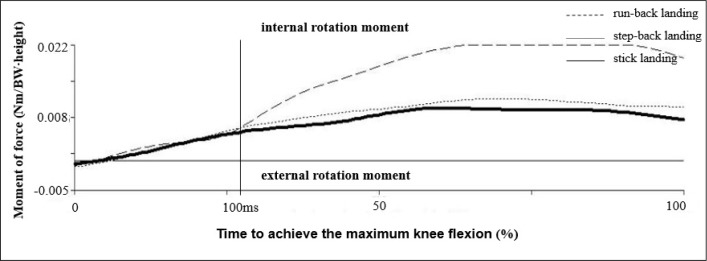
Internal-external rotation moment in the right knee joint (n=14)

In addition, it was proven that at the point when the athletes made contact with the ground while executing the run-back landing, they put their right lower limbs into internal rotation in the knee joint, which gradually transformed into external rotation. This is in contrast to stick landings and step-back landings, during which only external rotation in the knee joint on the right lower limb was observed (Figure 3). McLean et al. (2005) assumed that the increased abduction in women’s knee joints, when combined with an increased variability of tibia rotation, can contribute to an increased risk of ACL injuries. Myer et al. (2004) described a lower extremity motion pattern that was associated with the risk, such as ligament dominance in landing. Ligament dominance in landing is defined as landing with small knee flexion angle, significant medial knee motion related to femoral adduction and internal rotation, tibial external rotation, and high impact ground reaction forces. The findings also showed that there was a tendency during the execution of a run-back landing for internal rotation caused by the internal moment (Figure 4). Furthermore, the knee joint in case of the run-back landing creates external rotation of the knee while simultaneously experiencing the net moment of internal rotation by the action of passive structures of the knee joint. Such a combination of acting forces may expose the anterior cruciate ligament to undergo greater stress during the landing maneuver.

**Figure 5.**
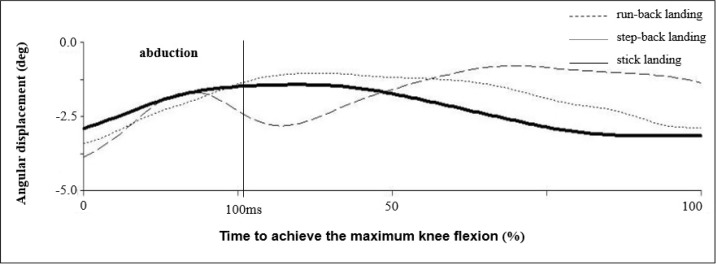
Abduction angle in the right knee joint (n=14)

The largest abduction angle upon contact with the ground was observed during the run-back landing. Moreover, the progress of the abduction angle in this type of landing shows a much greater variability compared to the stick landing and step-back landing (Figure 5). The run-back landing probably places higher demands on the neuromuscular awareness of the physical activity. Our hypotheses of higher demands on the neuromuscular awareness may be supported by lower VGRF standard deviation during the run back landing. From this point of view, it seems that normalization probably was not the reason for higher standard deviation of the valgus moment during a run back landing. Decreased neuromuscular control of the joint may place increased stress on the passive ligament structures (Li et al., 1999).

**Figure 6.**
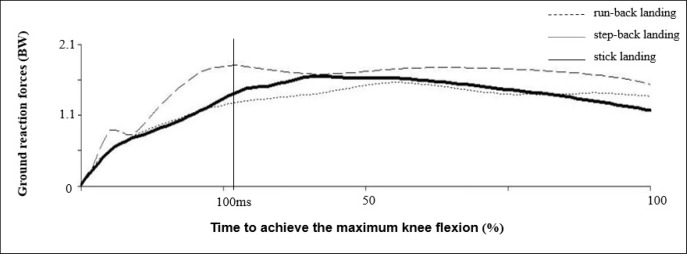
Vertical ground reaction forces (n=14)

From the perspective of ground reaction forces, the influence of the maximum values of the vertical reaction force was observed as quite significant in case of the run-back landing, as opposed to the step-back landing (Figure 6). Ground reaction force is understood as one of the risk factors for the incidence of ACL injuries (Yu et al., 2006). A step back after a block maneuver could reduce the vertical reaction forces and meet the requirements for a safe landing maneuver after a block in women’s volleyball.

## Conclusion

The highest valgus moment, which is considered to be a risk factor for ACL injuries, occurs during the run-back type of landing. The run-back landing also showed the highest vertical reaction forces. It is desirable that coaches teach players variations of landing. If a player has enough time in the situation of an unsuccessful block, a step-back landing should be used and subsequently the player could accelerate movement from the net. If a player does not have time to use a step-back landing then they should be trained to alternatively use their left and right lower extremities to carry out subsequent take-offs during their first steps from the net while performing the run-back landing to avoid possible chronic overloading of ACL.

## Figures and Tables

**Table 1:** Mean values and standard deviation of the normalized valgus moment (Nm/body weight·height) and normalized vertical ground reaction forces (body weight) on the right lower limb generated during the execution of three types of landing (n=14)

	Stick landing		Step-back landing		Run-back landing	

	*M*	*SD*	*M*	*SD*	*M*	*SD*
Valgus moment (Nm/BW·height)	0.014	0.007	0.015	0.010 ^a^	0.021	0.017^a^
Vertical ground reaction force (BW)	2.10	0.50	1.96	0.39	2.10	0.38

The same lettering located on the right following both mean and standard deviation values shows a statistically significant difference (p<0.05)
